# Panfolliculoma: Report of the Youngest Case and Literature Review of Its Histopathologic Variants

**DOI:** 10.3390/life12060881

**Published:** 2022-06-13

**Authors:** Shiow-Jen Juang, Khin-Than Win, Feng-Jie Lai

**Affiliations:** 1 Department of Dermatology, Chi Mei Medical Center, Tainan 71004, Taiwan; b00143@mail.chimei.org.tw; 2 Department of Pathology, Chi Mei Medical Center, Tainan 71004, Taiwan; a10905@mail.chimei.org.tw

**Keywords:** cystic panfolliculoma, panfolliculoma, trichofolliculoma, skin adnexal tumor, follicular neoplasm

## Abstract

**Background**: Panfolliculoma (PF) is a relative rare, benign follicular tumor comprised of all elements of the hair follicle, with a limited number of cases reported in the literature. Articles on the demographic and pathological analysis of this tumor are also lacking. **Case presentation**: In this report, we presented an unusual case of cystic PF on the back of a 14-year-old male, and we performed a thorough literature review and analysis of all previously reported cases. **Conclusions:** PF is a rare benign follicular neoplasm with characteristic differentiation toward all components of the hair follicle. In our analysis, PF occurred most frequently on the head region and was usually diagnosed in middle- to old-aged persons, with cystic PF being the most common histologic subtype. Since this tumor is rare and easily misdiagnosed as other tumors both clinically and pathologically, a thorough understanding of the histopathological manifestations and differential diagnosis of this tumor is necessary for both dermatologists and pathologists.

## 1. Introduction

Panfolliculoma (PF) is a benign follicular neoplasm with differentiation toward all components of the hair follicle, including both upper segment (infundibulum and isthmus) and lower segment (stem, bulb and hair matrix). To the best of our knowledge, since the first description by Ackerman et al. [1] in 1993, less than 50 cases of PF have been described in the medical literature [2,3,4,5,6,7,8,9,10,11,12,13,14,15,16,17,18,19]. 

Clinically, the tumor usually presents as a slow-growing nodule, resembling a basal cell carcinoma or cyst. Moreover, most of these cases occurred on the head region and were diagnosed after age 20. Based on the patterns on histopathology, Shan and Guo [11] classified PF into three subtypes: nodular, superficial and cystic. Additionally, a variant with endophytic architecture resembling a hair follicle was reported thereafter [15]. 

Here, we reported a rare case of cystic PF with unusual age at diagnosis, lesion size, clinical appearance and anatomic location. In addition to this case report, we also performed a comprehensive review of other reported cases of PF in an effort to summarize the characteristics of this tumor.

## 2. Case Report

**Figure 1:**
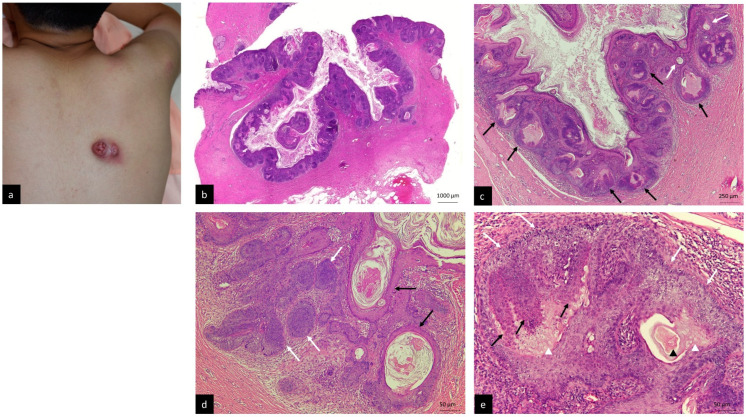
(**a**) A huge subcutaneous tumor with two overlying nodules and umbilicus-like openings on the back. (**b**) The well-circumscribed tumor showed a central keratin-filled cystic structure with opening to skin surface (H&E, 10×). (**c**) The cystic wall was composed of epithelial buddings of basaloid cells, hair-follicle-like structures (black arrows) and microcysts (white arrows) (H&E, 40×). (**d**) Microcysts lined by epithelial cells with granular layer and basket-wave corneocytes (black arrows); germinative cells with peripheral palisading (white arrows) (H&E, 200×). (**e**) Areas showing cells with eosinophilic trichohyalin granules (black arrows) and associated blue-gray corneocytes (white arrow heads); layers of clear cells (white arrows); eosinophilic shadow cells (black arrow heads) (H&E, 200×).

A 14-year-old male, without personal or family history, visited our dermatologic clinic with a huge skin tumor on his middle back which had been present for a few months, which had become painful and swelling for one week prior to his visit. Physical examinations revealed a large subcutaneous tumor, measuring 6 × 4 cm, with two overlying protruding, crusted and erythematous nodules with umbilicus-like openings (Figure 1a). Discharge from these umbilicus-like openings was also noted. An inflamed follicular cyst was diagnosed at first. Incision with drainage was performed several times and the result of bacterial culture was staphylococcus aureus. Oral antibiotics (cephalexin, doxycycline and clindamycin) and topical antibiotics (gentamicin cream and spersin ointment) were prescribed during the following months. The swelling and pain of the lesion gradually improved. Total excision of the tumor was arranged 6 months later after the first visit. 

Microscopic examination revealed a well-circumscribed, dermal-based neoplasm, which showed one dilated, pore-like, keratin-filled cystic structure (Figure 1b) with emanating lobules of basaloid cells, hair-follicle-like structures (Figure 1c, black arrows) and occasional microcysts (Figure 1c, white arrows). Lobular dermal aggregates of basaloid cells with multiple connections to the epidermis were also noted (Figure 1b). The intervening stroma showed fibroblast-like cells and lymphocyte infiltrates (Figure 1c). 

Within the wall of the main cystic structure, findings indicating differentiation into all the hair follicle components were detected: microcysts encircled by squamous epithelium with keratohyalin granules and corneocytes in a laminated and basket-weave array (Figure 1d, black arrows), representing infundibular differentiation; solid aggregates of follicular germinative cells with peripheral palisading (Figure 1d, white arrows); areas composed of cells with eosinophilic trichohyalin granules (Figure 1e, black arrows) and associated blue-gray corneocytes (Figure 1e, white arrow heads), indicating inner root sheath differentiation; cells with clear cytoplasm at the periphery of the trichohyalin-rich cells (Figure 1e, white arrows), resembling outer root sheath differentiation; and “shadow cells” resembling hair differentiation were also noted (Figure 1e, black arrow heads). Clefting artifact or myxoid stromal change were not evident.

**Figure 2:**
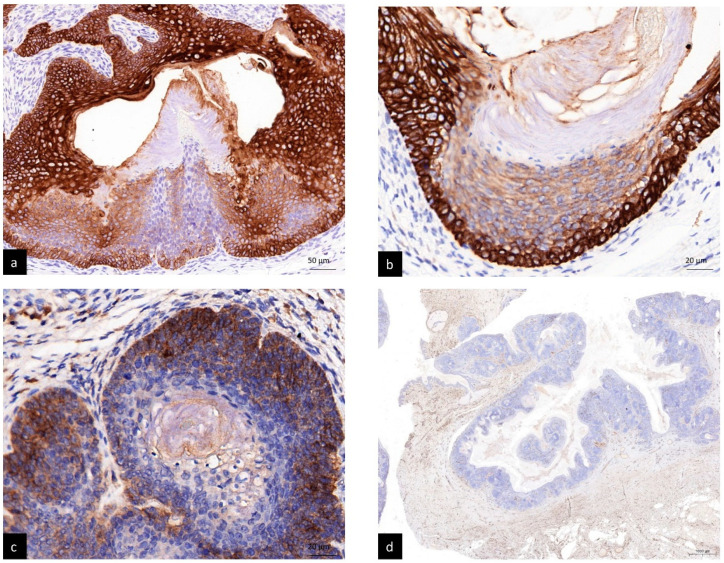
(**a**) 34βE12 stained most of the follicular structures in the tumor with infundibular, isthmus and outer root sheath differentiation, except for matrical cells (200×). (**b**) CK5/6 stain revealed similar results to 34βE12 stain (400×). (**c**) BerEP4 stained germinative cells (400×). (**d**) CD34 stained fibrotic stroma but negative in tumor cells (10×).

Immunohistochemical staining revealed that antibodies to 34βE12 (Figure 2a) and CK 5/6 (Figure 2b) stained most of the follicular structures in the tumor with infundibular, isthmus and outer root sheath differentiation, except for matrical cells, while BerEP4 staining highlighted germinative cells (Figure 2c). CD34 stained fibrotic stroma but not tumor cells (Figure 2d). 

## 3. Methods

A search of PubMed and Google Scholar was performed on 31 March 2022 using the keyword “panfolliculoma” to identify reported cases of PF. References were also reviewed for additional case reports not previously identified. Demographics, clinical impressions and accompanying histopathology were abstracted from the manuscripts. Empty data were excluded from the analysis. 

Summative data analysis was performed with the inclusion of the aforementioned case reports or case series included in the dataset. Data are presented as mean± standard deviation. 

## 4. Results

Review of the literature identified 18 manuscripts describing 43 individual cases of panfolliculoma, with the present case now accounting for a 44th patient (Table 1). Among these cases, one case reported in the manuscript by Schirren et al. in 1996 [2] had no clinical data and was excluded from our data analysis. Additionally, not all of the data points were possible to abstract from every case.

**Figure 3:**
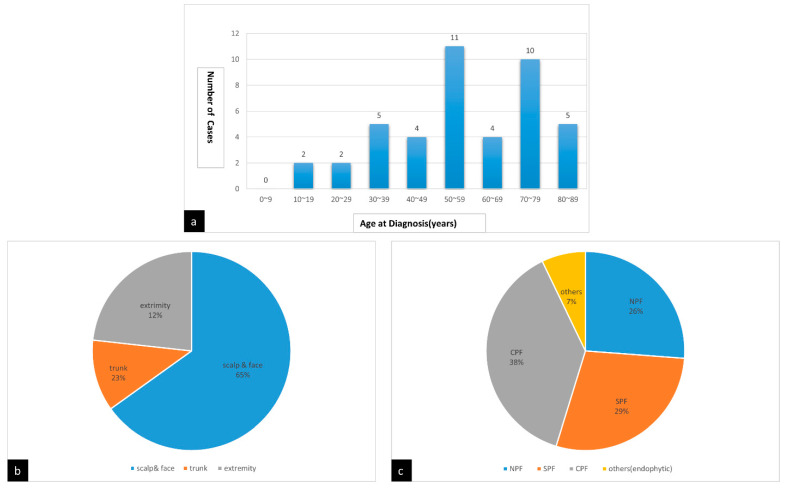
(**a**) Patient age at diagnosis. (**b**) Involved region of the body. (**c**) Subtypes of panfolliculoma.

Among 43 cases of PF enrolled for data analysis, the ages of patients ranged from 14 years old to 88 years old. However, a predisposition to occurrence in two age groups, i.e., between 50 and 59 years old (11/43) and between 70 and 79 years old (10/43), was noted (Figure 3a). The mean patient age at diagnosis was 56.6 ± 19.2 years. There was slight male predominance (Male:female = 1.4:1). Most of the lesions were located on the scalp and face region (65%), followed by extremities (23%) and trunk (12%) (Figure 3b).

Clinically, these lesions typically presented as slow-growing nodules, with their sizes ranging from 0.3 cm to 6 cm in diameter. Among 40 reported cases with available clinical impressions data, these tumors were usually clinically diagnosed as other benign or malignant skin lesions, with cyst (16/40), basal cell carcinoma (14/40) and squamous cell carcinoma (7/40) being the most frequent diagnoses. Other rare clinical impressions included wart, nevus, seborrheic keratosis, actinic keratosis, foreign body, comedone, cylindroma, poroma, lipoma, atheroma, angiolymphoid hyperplasia with eosinophilia and amelanotic melanoma (Table 1).

Based on the microscopic findings of 42 cases (1 case was excluded due to microscopic description being unavailable [3]), 11 cases (26%) were nodular subtype (NPF), 12 cases (29%) were superficial subtype (SPF), 16 cases (38%) were cystic subtype (CPF), and 3 cases (7%) were another variant (endophytic) (Figure 3c).

## 5. Discussion

PF is a unique, rare, benign follicular neoplasm. It presents with differentiation toward all parts of the hair follicle (infundibulum, isthmus, stem, bulb and hair matrix). According to the data we collected from the literature review, since Ackerman [1] first described these tumors in 1993, there were only 44 cases reported to date. PF occurs in both sexes with slight male predominance. The age distribution is wide, but with a tendency to occurrence in middle- and old-aged patients. Incidentally, our patient is the youngest case (14 years old) of PF cases reported in the literature so far. The tumor can be located on any skin site of the body, with scalp and face being the most commonly affected regions. It usually presents as a slow-growing nodule or mass, which is frequently clinically diagnosed as a cyst, basal cell carcinoma or squamous cell carcinoma. 

With respect to histopathological patterns, the classification of PF into three major subtypes was proposed by Shan and Guo [11]. These subtypes include nodular, superficial and cystic. Nodular PF (NPF) was defined as predominantly solid aggregates in the dermis; superficial PF (SPF) was defined as characteristic neoplastic aggregates within the epidermis and/or superficial dermis; and cystic PF (CPF) was defined as characteristic aggregates localized within or emanating from the cyst wall. We noticed that cystic subtype (43%) was the most common variant reported in the literature, which was followed by the superficial subtype (29%). This observation was different from that of the 19 cases reported by Shan and Guo [11], with superficial subtype (9/19) being the most common variant in their cases, followed by the cystic subtype (7/19). 

As the histologic findings described in our case, CPF exhibits a cystic structure located in the dermis, with the cyst wall lined by stratified squamous epithelium and cellular aggregates within or emanating from the cystic structure. The aggregates were composed of all components of the hair follicle, including follicular germinative cells, matrical cells and shadow cells, indicative of hair differentiation; cells with trichohyalin granules, indicative of inner root differentiation; clear cells resembling outer root differentiation; and different types of corneocytes. 

The histological differential diagnosis of CPF includes epidermal cyst, dilated pore of Winer, cystic trichoblastoma, pilomatricoma, pilar sheath acanthoma and trichofolliculoma. Among these skin lesions, trichofolliculoma is the main differential diagnosis of CPF. It also reveals differentiation toward all components of a hair follicle, but with more advanced and mature follicular differentiation than CPF. 

Although not essential for the diagnosis of PF, immunohistochemical staining highlights different components of the hair follicle and provides better identification of follicular differentiation. The 34βE12 antibody stains the infundibular and isthmic keratinocytes, CK 5/6 stains the outer root sheath and BerEP4 highlights germinative cells, while CD 34 stains cells with trichilemmal keratinization and perifollicular fibrous tissue [4,5]. However, in our case, CD34 stained fibrous stroma but not the tumor cells. This might be explained by the research performed by Poblet et al. [20], which pointed out that, in the typical anagen human follicle, CD34 immunoreactivity was detected only at the outermost layer of the outer root sheath, below the bulge zone, whereas the outer root sheath above the attachment zone of the arrector pili muscle was CD34-negative stained. In other words, not all of the hair follicle cells with outer root sheath differentiation had CD34-positive staining.

Fukuyama et al. [18] conducted an immunohistochemical analysis using a panel of antibodies to assess the global distribution of different hair follicle components within CPF, and attempted to provide insight into the pathogenesis. Expression of cytokeratin (CK)10, CK13, CK14, CK15, AE 13 and EpCAM within the lesion supported the differentiation of all epithelial lineages of the hair follicle. Fibroblastic dermal cells were distributed preferentially near CK15-negative epithelium or CK13-positive hair follicle-like structures, suggesting a role for epithelial–mesenchymal interactions in CPF pathogenesis.

Even if the pathogenesis of PF has been proposed, we do not know enough about whether PF is hereditary. As in our case, most of the published cases of PF had no family history. However, Estrada-Castañón et al. [17] reported a female patient with similar lesions in the same location as her daughter. Further genetic studies should help us understand whether there is a genetic predisposition to the disease.

In conclusion, PF is a rare, benign follicular neoplasm with characteristic differentiation toward all components of the hair follicle. The tumor frequently occurs in the head region and is usually diagnosed in middle- and old-aged persons, with a slight male predominance. Among these tumors, cystic PF is the most common subtype. Immunohistochemical analysis of the tumor not only provides better identification of follicular differentiation, but also provides a hint about the pathogenesis of this unique tumor.

## Figures and Tables

**Table 1:** The Clinical and Histopathologic Parameters of 44 case of PF.

Author, Year	Case Number	Age	Sex	Tumor Location	Clinical Impression	Lesion Size (cm)	Histopathology Variants
Schirren et al. 1996 [2]	1	53	M	infraclavicular	follicular cyst	2~3	NPF
	2	N/A	N/A	N/A	N/A	N/A	N/A
	3	22	F	scalp	cylindroma	2~3	NPF
	4	49	F	right eyebrow	BCC	2~3	NPF
Marini et al. 2006 [3]	5	46	F	nostril	N/A	N/A	N/A
Hoang et al. 2006 [4]	6	33	F	scalp	cyst	3 × 2 × 2	CPF
Huang et al. 2010 [5]	7	41	F	scalp	EC, pilar cyst	N/A	NPF
	8	51	M	left eyebrow	ALHE, leiomyoma	N/A	NPF
Harris et al. 2011 [6]	9	81	M	right medial thigh	inflammed SK	0.6	SPF
	10	61	F	rightl lateral thigh	SCC	0.3	SPF
Ruiz-Villaverde et al. 2011 [7]	11	56	M	Right tibia	EC	<3	NPF
Kacerovska et al. 2012 [8]	12	53	M	occipital scalp	atheroma	N/A	NPF with sebaceous differentiation
Idriss et al. 2013 [9]	13	55	M	right leg	N/A	0.8	SPF
Alkhalidi et al. 2013 [10]	14	19	F	scalp	pilar cyst, lipoma	0.9 × 0.8	CPF
Shan et al. 2014 [11]	15	76	F	Left forearm	BCC	0.5 × 0.4	SPF
	16	65	M	Left cheek	SK, AK, BCC	0.4 × 0.3	SPF
	17	79	M	Left nasal ala	BCC, dermal cyst	0.6 × 0.5	CPF
	18	32	M	Left scalp	Sebaceous cyst	0.6 × 0.5	CPF
	19	55	M	Right leg	VV	0.5 × 0.4	SPF
	20	82	M	Upper back	BCC	1.2 × 0.8	SPF
	21	53	M	Right low lip	Cyst	0.3 × 0.3	CPF
	22	70	F	Right lateral forehead	SCC, BCC	0.8 × 0.6	CPF
	23	22	F	Scalp	Cyst	0.7 × 0.6	CPF
	24	33	F	Left parietal scalp	BCC, nevus	0.8 × 0.8	NPF
	25	49	M	Right nostril	Cyst, BCC	0.5 × 0.4	CPF
	26	70	M	Central forehead	SCC, BCC	0.7 × 0.6	SPF
	27	72	M	Left post auricular	Wart, cyst	0.4 × 0.4	SPF
	28	59	F	Right lateral leg	Wart	0.4 × 0.3	SPF
	29	71	M	Forehead	BCC	0.4 × 0.3	SPF
	30	83	F	Left scalp	SCC	4 × 3	CPF
	31	87	M	Right temple	SCC, BCC	0.8 × 0.7	NPF
	32	70	M	Nose	Cyst, BCC	0.4 × 0.3	NPF
	33	67	M	Right lower leg	SCC	0.4 × 0.3	SPF
Patel et al. 2014 [12]	34	73	M	Left helix	EC	N/A	CPF
Neill et al. 2016 [13]	35	64	F	right forearm	SCC, amelanotic melanoma, poroma	0.8	CPF
Nishikawa et al. 2016 [14]	36	36	F	back	N/A	2.3 × 3	NPF
Terushkin et al. 2016 [15]	37	56	F	Left cheek	BCC	N/A	endophytic
	38	88	M	right anti-helix	cyst	N/A	endophytic
	39	53	F	right pretibial leg	foreign body	N/A	endophytic
Parvinnejad et al.2017 [16]	40	74	M	abdomen	BCC	N/A	CPF
Estrada-Castañón et al. 2017 [17]	41	55	F	eyelid	comedone	N/A	CPF
Fukuyama et al. 2017 [18]	42	70	M	occipital scalp	trichilemmal cyst	2.4	CPF
Rivera et al.2018 [19]	43	35	M	right occipital scalp	pilar cyst	1.3	CPF
Present case	44	14	M	back	follicular cyst	6 × 4	CPF

BCC, basal cell carcinoma; EC, epidermal cyst; ALHE, angiolymphoid hyperplasia and eosinophilia; SK, seborrheic keratosis; AK, actinic keratosis; VV, verruca vulgaris; N/A: not available.
